# The Gut–Heart Axis and Its Role in Doxorubicin-Induced Cardiotoxicity: A Narrative Review

**DOI:** 10.3390/microorganisms13040855

**Published:** 2025-04-09

**Authors:** Carolina Rodrigues Tonon, Amanda Gomes Pereira, Natália Fernanda Ferreira, Marina Gaiato Monte, Nayane Maria Vieira, Anderson Seiji Soares Fujimori, Paola da Silva Ballin, Sergio Alberto Rupp de Paiva, Leonardo Antonio Mamede Zornoff, Marcos Ferreira Minicucci, Bertha Furlan Polegato

**Affiliations:** Internal Medicine Department, Botucatu Medical School, Sao Paulo State University (UNESP), Botucatu 18618-687, Brazilbertha.polegato@unesp.br (B.F.P.)

**Keywords:** gut microbiota, cardiac remodeling, cardiovascular disease, short-chain fatty acids, tight junctions, LPS

## Abstract

Doxorubicin is a widely used chemotherapy for the treatment of several types of cancer. However, its application is restricted due to adverse effects, particularly cardiotoxicity, which can progress to heart failure—a chronic and debilitating condition. Several mechanisms have been identified in the pathophysiology of doxorubicin-induced cardiotoxicity, including oxidative stress, mitochondrial dysfunction, inflammation, and disruption of collagen homeostasis. More recently, dysbiosis of the gut microbiota has been implicated in the development and perpetuation of cardiac injury. Studies have reported alterations in the composition and abundance of the microbiota during doxorubicin treatment. Therefore, as of recent, there is a new field of research in order to develop strategies involving the gut microbiota to prevent or attenuate cardiotoxicity since there is no effective therapy at the moment. This narrative review aims to provide an update on the role of gut microbiota and intestinal permeability in the pathophysiology of cardiovascular diseases, and more specifically doxorubicin-induced cardiotoxicity. Additionally, it seeks to establish a foundation for future research targeting gut microbiota to alleviate cardiotoxicity.

## 1. Introduction

Cancer remains a leading cause of morbidity and mortality and represents a public health issue. In 2025, it is estimated that there will be 2,041,910 new cases of cancer in the United States, resulting in approximately 618,120 deaths, which equates to roughly 1693 deaths per day [1].

Anthracyclines have been employed for over 40 years as a class of chemotherapy agent in the treatment of various solid tumors, such as breast, lung, thyroid, osteosarcoma and hematologic malignancies. Doxorubicin, an anthracycline, is often the first line treatment in numerous scenarios due to its high efficacy compared to regimens lacking this medication [2]. However, the use of doxorubicin is limited due to several side effects, particularly cardiotoxicity, as well as gastrointestinal, bone marrow, liver and kidney toxicities [3,4].

Doxorubicin is an antibiotic produced by the bacteria *Streptomyces peucetius* [2]. The cytotoxic properties were first identified in 1950, and it received approval for clinical use in 1963. The antineoplastic action of doxorubicin involves two primary mechanisms. The first is related to the pharmacology of the drug, with inhibition of topoisomerase II, an enzyme that acts on DNA replication. The second mechanism involves the production of reactive oxygen and nitrogen species, leading to oxidative stress that can adversely affect cellular membranes, proteins and DNA, ultimately triggering cell death via multiple pathways [5,6,7,8].

Cardiotoxicity is the most serious and concerning collateral effect as it can lead to heart failure, a chronic and irreversible disease that severely impairs the heart’s ability to efficiently pump blood to organs and tissues [9]. The condition has a high mortality rate and is a significant burden to patients and healthcare systems. Currently, the recommendations to prevent cardiotoxicity are limited; prophylactic use of angiotensin-converting enzyme inhibitors (ACE-Is) or angiotensin receptor blockers (ARBs) and beta-blockers may be considered to reduce the development of cardiotoxicity. Dexrazoxane is the only medication approved for primary prevention of doxorubicin-induced cardiotoxicity, but only in selected patients [10], and with some collateral effects, including hematological toxicity and liver dysfunction [11]. Nevertheless, no other treatments have been approved to prevent toxicity in other different clinical scenarios.

In addition to cardiac effects, chemotherapy also adversely impacts the gastrointestinal tract (GIT), contributing to a decline in quality of life, decreased treatment adherence, and increased healthcare costs. Chemotherapy agents affect healthy cells indiscriminately, with enterocytes being particularly susceptible due to their rapid turnover. Beyond the conventional mechanisms of toxicity discussed throughout this narrative review, doxorubicin can also induce gastrointestinal toxicity by disrupting the gut microbiota (GM). Doxorubicin may compromise the intestinal barrier and facilitate the translocation of endotoxins and microbial elements into systemic circulation. These translocated elements can trigger systemic inflammation, contributing to the development, amplification and maintenance of cardiovascular diseases [12,13,14].

Alterations in the gut microbiota have been observed in cardiovascular diseases (CVD) and chronic metabolic disorders, such as obesity, dyslipidemia, insulin resistance, atherosclerosis and heart failure [15,16,17]. DNA from several species of bacteria from the gut were found at atherosclerotic plaques, suggesting that the gut microbiota may participate in the physiopathology of coronary artery disease. Additionally, trimethylamine N-oxide (TMAO), a metabolite produced by gut microbiota, has been positively associated with the presence of atherosclerosis [18,19].

Moreover, a less diverse and less rich microbiota has been documented in spontaneous hypertensive rats and in patients with high blood pressure. In addition, a more pathogenic microbiota and more permeable gut have been observed in patients with chronic heart failure (CHF) compared to healthy individuals [20,21,22,23]. Finally, cardiovascular diseases have been associated with increased intestinal permeability, as evidenced by increased lipopolysaccharides (LPS) in conditions such as hypertension [24], chronic heart failure [25], myocardial infarction [26] and atherosclerosis [27].

Recently, the role of microbiota in chemotherapy-induced toxicity has gained attention from researchers. Some studies have indicated that chemotherapy can alter and reduce microbiota diversity. For example, Montassier et al. reported a decrease in the abundance of *Bacillota* (formerly *Firmicutes*) and *Actinomycetota* (formerly *Actinobacteria*), while *Pseudomonadota* (formerly *Proteobacteria*) was higher in the feces of lymphoma patients undergoing chemotherapy [28,29]. Similarly, Motoori et al. reported a decrease in *Lactobacillus* and *Clostridium difficile*, while *Enterococci* was higher after a combined regimen of chemotherapy [30]. Lastly, Galloway-Peña et al. showed a decreased microbiota diversity in patients with acute myeloid leukemia after chemotherapy induction [31].

These findings suggest that chemotherapy can alter the composition of gut microbiota and impair intestinal permeability, which can potentially contribute to the development and perpetuation of cardiotoxicity. Therefore, protecting the gut microbiota may represent a promising strategy to prevent or attenuate the complications of chemotherapy in both the gastrointestinal tract and the cardiovascular system given the established associations between the microbiota, intestinal permeability and cardiovascular diseases [12,29,32,33].

This narrative review aims to provide an updated review of the mechanisms involved in doxorubicin-induced cardiotoxicity and to more extensively discuss recent findings regarding the association between dysbiosis and DOX-induced cardiotoxicity. Focusing on the gut microbiota may offer a potential strategy to reduce cardiac toxicity, making it a promising area for future research.

## 2. Methods

We performed a comprehensive search across PubMed, Scopus, ScienceDirect and Web of Science, completing the final search in March 2025. The selection included international, English-language articles. The search strategy combined the keywords “doxorubicin-induced cardiotoxicity” or “cardiovascular diseases” with terms related to microbiota, microorganisms, short-chain fatty acids, and the intestinal barrier. After removing duplicates, the remaining abstracts were screened to ensure they met the review’s inclusion criteria. Eligible studies focused on the effects of cardiovascular diseases, particularly doxorubicin-induced cardiotoxicity, on gut microbiota and the intestinal barrier, as well as potential therapeutic approaches to mitigate cardiovascular damage. The selected studies were then summarized and synthesized into the narrative review.

## 3. Doxorubicin-Induced Cardiotoxicity

Doxorubicin-induced cardiotoxicity can present with a variety of signs and symptoms including arrhythmias, pericarditis, myocarditis, ventricular dysfunction and heart failure. The induced cardiotoxicity is dose-dependent, where the risk increases as the dosage increases [34,35]. Also, other factors contribute to the risk of cardiotoxicity, including patient age, duration of infusion, prior radiotherapy and preexisting cardiovascular diseases [36].

Multiple interconnected mechanisms are involved in the pathophysiology of doxorubicin-induced cardiotoxicity. The primary mechanisms include direct DNA damage, oxidative stress, inflammation, mitochondrial dysfunction, calcium handling alterations, a reduction in contractile proteins and collagen homeostasis disruption [37,38,39,40,41,42,43].

Classically, oxidative stress (OS) is widely recognized as a central factor in the development of cardiotoxicity. OS is defined as an increased production of reactive oxygen and nitrogen species coupled with diminished antioxidant activity. Doxorubicin increases the production of reactive nitrogen and oxygen species while reducing the activity of antioxidants enzymes, which are already lower in cardiac tissue, making the organ more susceptible to injury [44]. This imbalance leads to protein and lipid oxidation, DNA damage, mitochondrial dysfunction, exacerbation of inflammatory pathways and alterations in the homeostasis of the extracellular matrix [37,38,39,40,41,42,43]. Antioxidant substances, including ascorbic acid, N-acetylcysteine, coenzyme Q10, hydropersulfides donors, among others, have been studied in the context of doxorubicin-induced cardiotoxicity, with variable results, but to date, there are no medications approved to reduce oxidative stress in the context of doxorubicin-induced cardiotoxicity [11].

Furthermore, inflammation plays a critical role in the progression of cardiac dysfunction. Inflammation is a complex biological response intended to eliminate harmful stimuli and repair damaged tissue; however, excessive and prolonged inflammation can result in tissue destruction and progressive fibrosis. While fibrosis serves to preserve tissue architecture, it can also become pathological and impair organ function [45].

The inflammatory process is initiated by the activation of nuclear factor kappa B (NF-κB), a transcription factor [46] that induces the release of pro-inflammatory cytokines, including tumor necrosis factor alpha (TNF-α), interleukin (IL)-6, interleukin (IL)-1β, angiotensin II, and the recruitment of macrophages and monocytes [47,48,49,50]. These cytokines activate fibroblasts into myofibroblasts, which are responsible for the synthesis of the extracellular matrix and cardiac fibrosis [42,45,51,52,53,54].

Mitochondrial dysfunction has also been extensively studied in the context of doxorubicin-induced cardiotoxicity. Doxorubicin has a strong affinity for cardiolipin, which is abundant in the inner mitochondrial membrane. This interaction disrupts the mitochondrial electron transport chain, resulting in increased oxidative stress and creating a vicious cycle that activates apoptosis and necrosis [36,42]. Moreover, doxorubicin impairs mitochondrial biogenesis, including fusion and fission processes, which are essential for cellular survival [42].

Other mechanisms implicated in cardiotoxicity include alterations in matrix metalloproteinases (MMPs) and tissue inhibitors of metalloproteinases (TIMPs), dysregulation in calcium homeostasis and reduction in contractile proteins. Doxorubicin may inhibit calcium pumps by altering the expression of muscle genes, such as the sarcoendoplasmic reticulum ATPase gene (SERCA2a) and the phospholamban gene [42,55]. However, these mechanisms are not discussed extensively, as they are not the focus of this narrative review.

All these mechanisms culminate in a final pathway known as cardiac remodeling, which is defined as molecular, cellular and interstitial changes in the heart. These manifest as alterations in cardiac size, shape and function, and ultimately the development of cardiac dysfunction [8,9,12,13,14,56].

The final consequence of cardiac remodeling is cardiac dysfunction, which may initially be asymptomatic but can progress to ventricular dysfunction with the typical signs and symptoms of heart failure (HF) [57]. Common symptoms of HF include shortness of breath, ankle swelling and fatigue, often accompanied by clinical signs such as elevated jugular venous pressure, pulmonary crackles and peripheral edema [58].

HF affects an estimated 64 million people worldwide, and its prevalence is projected to rise due to the aging of the population [59]. Despite improvements in survival rates with modern therapies, the prognosis remains poor, with elevated mortality rates [59]. Approximately 50% of patients diagnosed with cardiac dysfunction die within five years, and 40% of patients die within one year of hospitalization for heart failure [57].

Deaths resulting from cardiovascular diseases, including heart failure, significantly exceed those from all types of cancer. This is the main reason why doxorubicin-induced cardiotoxicity is an extremely important issue.

In summary, doxorubicin-induced cardiotoxicity is a complex and multifactorial process, wherein some mechanisms exacerbate others, ultimately leading to a common outcome: cardiac remodeling, cardiac dysfunction and finally heart failure. This causes a huge burden to patients and to healthcare providers.

## 4. Microbiota, Intestinal Permeability and Cardiovascular Diseases

As previously mentioned, alterations in the composition of the gut microbiota are associated with cardiovascular diseases (CVDs) and chronic metabolic disorders [15,16,17]. The microbiota is a complex population of microorganisms, including bacteria, fungi, viruses and protozoans, that coexist symbiotically on human surfaces and in body cavities, such as the skin and mucosa [60]. It is estimated that the human microbiota contains approximately 10^14^ bacteria, which is roughly the number of cells in our body [61]. The gastrointestinal tract houses the greatest number and diversity of microorganisms, primarily due to its extensive surface area (200 m^2^) and the presence of nutrients, which are essential for microbial survival. It is estimated that the colon contains more than 70% of all microorganisms in the human body [62]. The most common bacterial phyla in the human intestines include *Bacillota* (formerly *Firmicutes*), *Bacteroidota* (formerly *Bacteroidetes*), *Actinomycetota* (formerly *Actinobacteria*) and *Pseudomonadota* (formerly *Proteobacteria*) [63].

The intestinal microbiota is responsible for several vital functions in the human body, including the breakdown of non-digestible macromolecules, such as carbohydrates and proteins into short chain fatty acids (SCFAs), which are easily absorbed in the distal intestine. Additionally, it is involved in vitamin synthesis, preventing pathogenic bacteria colonization, drug modification, regulating epithelial cell proliferation and differentiation, producing substances that act in the metabolism of intestinal epithelial cells and immunomodulation [60,62,64,65,66].

Consequently, the gut microbiota (GM) establishes a complex relationship with the intestinal surface, modulating its integrity through several mechanisms. GM stimulates the production of α-defensins by Paneth cells, which are antimicrobial peptides that play a role in host defense, protecting the mucosa by competing with pathogenic microorganisms, modulating myelopoiesis, interfering with immune cell recruitment and producing SCFAs [67,68,69]. SCFAs are the primary energy source for colonocytes and suppress the production of pro-inflammatory mediators, such as interleukin-6 (IL-6), tumor necrosis factor alpha (TNF-α) and nitrous oxide (NO), while stimulating the release of the anti-inflammatory mediator interleukin-10 (IL-10) [70]. They also promote the production of mucins [71,72] and reassemble tight junctions by upregulating proteins essential for maintaining intestinal integrity [63,73,74,75].

Under normal conditions, the microbiota coexists harmoniously with the host. However, when this homeostasis in disrupted, dysbiosis occurs, characterized by alterations in gut microbiota function and composition, which can adversely affect intestinal permeability [76].

The intestinal barrier is a multilayered system consisting of a complex structure of epithelial cells from the luminal environment that prevent excessive water and electrolyte loss as well as microorganisms entering the body [77]. The physical barrier is composed of four layers of epithelium (mucosa, submucosa, muscularis and serosa) with a mucus layer. Adjacent to the physical barrier is the chemical barrier, which includes immune molecules, cytokines, antimicrobial peptides, inflammatory mediators and the microbiota. Epithelial cells are interconnected by tight junctions (TJ), composed of several intra-membrane proteins, such as occludin, claudins and zonula occludens (ZOs) 1, 2 and 3, which regulate the flow of water and ions. Beneath the TJs are the adherence junctions (AJs), composed of catenins and cadherins, with the desmosomes underneath [78].

Historically, intestinal permeability was assessed using orally ingested solutes such as mannitol and lactulose; however, these methods are time-consuming. Consequently, biomarkers expressed by intestinal cells and/or released upon mucosa damage such as LPS and LPS-binding protein (LBP), structural proteins of the tight junctions (claudin, occludin) and other biomarkers such as intestinal fatty acid-binding protein (I-FABP) have gained prominence [79]. Zonula occluden proteins, collectively referred to as zonulins, can reversibly open the tight junction protein complex, thereby modulating intestinal permeability and serving as endogenous modulators of TJ [80]. Recent studies have utilized serum concentration of zonulins to determine intestinal permeability with greater accuracy; however, serum zonulins dosage should be used with caution, since most used enzyme-linked immunosorbent assays (ELISAs) identify complement C3 and properdin rather than zonulin itself [81].

Disruption of the intestinal barrier allows the translocation of endotoxins and microbial products, exposing these substances to immune cells. Recognition of these substances is mediated by pattern recognition receptors (PRRs) expressed on immune cells, commonly known as toll-like receptors (TLRs). The interaction between TLRs and microorganisms occurs via pathogen-associated molecular patterns (PAMPs), such as lipopolysaccharides (LPSs) and proteoglycans [82]. Upon activation, TLRs induce the release of inflammatory cytokines and the maturation of dendritic cells, leading to the activation of a systemic inflammatory response. This systemic inflammatory response has already been implicated in the development and progression of heart failure [15,16,83]. Additionally, LPS has been shown to induce apoptosis in cardiomyocytes in vivo [84] and to increase the production of mitochondrial reactive species, which are also involved in fibrosis and hypertrophy of cardiomyocytes [85].

The earliest evidence linking inflammation to heart failure dates back to 1990 when Levine et al. showed elevated values of pro-inflammatory cytokines in patients with heart failure compared to healthy subjects [86]. Several pro-inflammatory cytokines and inflammatory factors have been shown to participate in the pathogenesis of heart failure [87]. Since then, various studies have highlighted the role of inflammation in the development and progression of both acute and chronic heart failure [88]. TNF-α, the most studied cytokine in this context, induces cardiomyocyte hypertrophy, activates matrix metalloproteinases, inhibits matrix metalloproteinases inhibitors, activates NF-κB leading to neutrophil migrations and stimulates ROS production [89,90,91]. There is substantial evidence that, regardless of the etiology of heart failure, cytokines and chemokines contribute to cardiac dysfunction and remodeling [92].

In heart failure, inflammation is activated and maintained by the toll-like receptors (TLRs). There are ten types of TLRs in humans, but only three types are present in myocytes: TLR-2, TRL-3, TRL-4. Activation of these receptors leads to the activation of NF-kB, driving inflammation [93].

The importance of inflammatory cells in cardiovascular diseases, especially macrophages, monocytes and neutrophils, has been well established. Several inflammatory mediators, such as IL-1, IL-6 and TNF-α, are implicated in this process. Elevated concentrations of IL-1 stimulate the secretion of other cytokines, enhance the expression of adhesion molecules, promote the proliferation of endothelial and smooth muscle cells, activate macrophages and increase vascular permeability. In addition, inflammatory cells, along with TNF-α, promote the secretion of proteins which regulate the inflammatory cascade. This cascade transforms fibroblasts and other inflammatory cells into myofibroblasts, the most important cells responsible for ECM synthesis, leading to increased ECM production [45].

Various strategies have been explored to mitigate cardiac inflammation. Infliximab, an immunoglobulin targeting TNF- α, was assessed in patients with a reduced ejection fraction, but no clinical improvement was demonstrated [94]. IL-1 inhibition with canakinumab has been shown to reduce hospitalization due to heart failure in patients with prior myocardial infarction [95]. In rats with hypertension-induced heart failure, colchicine, an anti-inflammatory drug, improved diastolic function, reduced inflammatory infiltration in cardiac tissue, and decreased the expression of TNF-α, chemokine ligant-2 (CCL2), monocyte chemoattractant protein-1 (MCP1), NLRP3 and NF-κB in the myocardium [96]. Despite some advances in this area, targeting inflammation to manage heart failure remains an enigma.

Although systemic inflammation is the primary pathophysiological mechanism proposed for the development of cardiovascular diseases related to gut microbiota dysfunction, more recently, other mechanisms have emerged. SCFAs, particularly butyrate, propionate, and acetate, in addition to acting in the maintenance of intestinal barrier integrity and having anti-inflammatory effects as cited previously, appear to regulate vascular tonus, causing vasodilation and lowering blood pressure [97]. Propionate has shown to attenuate cardiac hypertrophy, fibrosis and vascular dysfunction in hypertensive mice and butyrate has shown to reduce blood pressure and heart rate after administration in the colon of rats [98].

## 5. Doxorubicin-Induced Cardiotoxicity and Inflammation

Inflammation has been shown to contribute to the development and progression of doxorubicin-induced cardiotoxicity. In diverse experimental models, doxorubicin consistently showed increased levels of inflammatory mediators as reported in the following studies. In cardiomyocyte cells treated with doxorubicin, Hu et al. and He et al. reported increased IL-1β and TNF-α [99,100]; Peng et al. noted upregulation of IL-1β and monocyte chemoattractant protein-1 (MCP-1) and increased activation of the NF-κB pathway [101]; and finally, Jiang et al. observed elevated concentrations of TNF α, IL-1β and IL-6 [102]. In rat models, Wan et al. and Fang et al. showed increased levels of IL-1β, IL-18, and TNF-α [103,104] while Akolkar et al. showed significant increase in NF- κB [105].

Moreover, doxorubicin appears to affect gut microbiota and intestinal cells. Huang et al. [106] identified a difference in beta-diversity of gut microbiota of mice treated with doxorubicin, while Lin et al. [107] showed lower bacterial species richness in rats receiving the drug. An et al. [13] revealed that doxorubicin exacerbates intestine damage by increasing the incidence of colonic ulcers and goblet cell loss, promoting lymphocyte cluster infiltration and decreasing the expression of the tight junction protein ZO-1, along with alterations in the gut microbiota richness. Adding to this, Wu et al. [108] showed that doxorubicin induced a distinct gut bacterial community compared to control rats, disrupted the epithelial barrier, increased crypt depth, decreased the number of goblet cells and decreased levels of ZO-1 and occludin. Lastly, Cray et al. reported disorganization of occludin and ZO-1 in human colon culture cells treated with doxorubicin [109].

**Figure 1:**
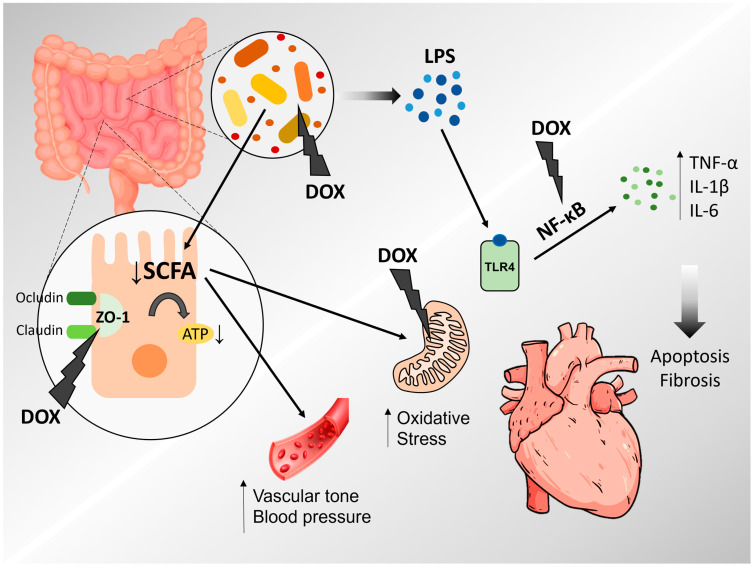
Interplay between gut microbiota and doxorubicin-induced cardiotoxicity. Figure made by the authors in Power Point, Microsoft.

Therefore, by affecting the gut microbiota and intestinal permeability, doxorubicin may propagate and sustain an exacerbated systemic inflammatory response, thereby triggering several pathways associated with cardiac remodeling and dysfunction (Figure 1).

## 6. Interventions to Target Gut Microbiota in Cardiovascular Diseases

Several studies have investigated the gut microbiota as a target to attenuate cardiovascular diseases such as hypertension, diabetes, dyslipidaemia, chronic kidney disease, myocardial infarction and heart failure, as cited subsequently.

In the context of hypertension, some clinical trials have evaluated the use of substances and their effects on blood pressure. For instance, Xue et al. reported alterations in the gut microbiota, specifically an increase in the abundance of *Bifidobacterium* and *Trichosporium*, along with improved blood pressure in hypertensive patients following the administration of oat bran, a dietary fiber [110]. Taladrid et al. reported that a grape-derived substance, administered for six weeks, led to reductions in both systolic and diastolic blood pressure in high-risk cardiovascular patients [111]. This was further correlated with changes in the abundance of certain gut microbiota species and a decrease in propionic acid levels. A metanalysis revealed a significant decrease of 3.56 mmHg in systolic and 2.38 mmHg in diastolic pressure in patients who received probiotics [112]. Furthermore, in experimental models, interventions such as fecal transplantation, antibiotics, prebiotics, probiotics, vitamin C, dietary fibers, natural compounds and SCFA have been shown to modify microbiota composition and improve blood pressure [113,114,115,116,117,118,119].

Regarding diabetes, Deng et al. reported that Tibetan tea, a fermented dark tea, decreased oxidative stress and the pro-inflammatory cytokines TNF-α and IL-6 while increasing the anti-inflammatory cytokine IL-4, along with restoring the β-diversity of gut microbiota in a model of type 1 diabetes in mice [120]. Additionally, Zeng et al. showed that epicatechin, a polyphenol, reduced oxidative stress, the abundance of LPS-producing bacteria and serum LPS in rats with type 2 diabetes [121].

In the context of dyslipidaemia, theabrownin, a component of dark tea, was found to reduce the serum concentrations of TNF-α, IL-1β and IL-6. Additionally, it increased the species richness and diversity of the gut microbiota, as well as elevated fecal acetic acid in hamsters with high-fat diet-induced dyslipidaemia [122]. Furthermore, in a randomized clinical trial, Salamat et al. reported that patients with dyslipidaemia who received probiotic supplementation exhibited decreased serum endotoxin and TMAO, a metabolite produced by gut microbiota metabolism [123].

Regarding interventions targeting the microbiota to improve heart failure, Lu et al. showed improvement in cardiac remodeling and modulation of gut microbiota in rats treated with qiliqiangxin, a herbal compound commonly used in Chinese medicine [124]. In addition, Gan et al. reported an attenuation of cardiac remodeling after probiotic administration in rats post infarction; however, they did not observe significant changes in gut microbiota [125]. Furukawa et al. found that β-conglycinin, a soy protein, enhanced systolic parameters and reduced cardiac hypertrophy, along with an increase in SCFA-producing intestinal bacteria and fecal SCFAs [126].

## 7. Interventions on DOX-Induced Cardiotoxicity by Targeting the Gut Microbiota and Intestinal Permeability

Several substances and medications were tested experimentally aiming to attenuate doxorubicin-induced cardiotoxicity. Natural compounds like flavonoids, alkaloids, polyphenols, anthraquinones, polysaccharides, as well as vitamins, hormones, exosomes and marketed drugs such as doxycycline, calcium channel blockers, statins, sodium-glucose cotransporter 2 inhibitors, renin–angiotensin–aldosterone system inhibitors and beta-blockers showed beneficial effects on cardiotoxicity induced by doxorubicin [10,127,128,129].

However, there is a limited number of studies specifically highlighting the interplay between gut dysbiosis and cardiac dysfunction following doxorubicin treatment [13,130]. In this context, few strategies have been explored as an attempt to mitigate intestinal damage by enhancing the diversity and richness of the gut microbial community, thereby preventing or attenuating cardiotoxicity.

Recent interest has focused on dietary compounds, particularly those with antioxidant and anti-inflammatory properties, as well as approaches that directly modulate the microbiota.

Table 1 summarizes studies addressing the modulation of gut microbiota and cardioprotective effects in DOX-treated animals.

### 7.1. Polyphenols

Polyphenols represent the largest group of phytochemicals found mainly in plant-based foods and are associated with the prevention of several degenerative conditions, particularly cardiovascular diseases [137]. It is well established that polyphenols can modulate the gut microbiota [138]. From this perspective, an experimental study evaluated the effect of purified polyphenols from Burdock (*Arctium lappa* L.—ALPP) in microbiota composition on DOX-induced heart failure [131]. A 3-week pretreatment with ALPP promoted a decrease in oxidative stress, inflammatory indexes, and activities of casein kinase and lactate dehydrogenase, which was related to the alleviation of cardiotoxicity induced by cumulative DOX medication. In addition, an overall amelioration of intestinal homeostasis and integrity was observed in groups that received the pretreatment with polyphenols. ALPP consumption substantially improved the production of short-chain fatty acids (SCFAs) after DOX treatment, which is considered the main source of energy for colonocytes and a protective agent against pathogens in the intestinal environment. Moreover, pretreatment with ALPP was able to directly modulate the gut microbiota by promoting the growth of probiotic bacteria such as *Lactobacillaceae*, *Ruminococcaceae*, and *Roseburia.* Additionally, the treatment reduced the abundance of both *Proteobacteria*, often considered a marker of dysbiosis of gut microbiota, and opportunistic pathogens such as *Enterococcus*, *Erysipelatoclostridium* and *Escherichia*/*Shigella*, related to the leak of the intestinal barrier and increased systemic inflammation.

Similarly, a recent study conducted by Lin et al. [107] evaluated the anticardiotoxic effects of yellow wine polyphenolic compound in chronic DOX-treated rats. The study showed a clear connection between modulation of gut microbiota and attenuation of DOX-induced cardiotoxicity. The authors demonstrated that 8 weeks of pretreatment with this yellow wine polyphenolic compound significantly ameliorated DOX-mediated cardiotoxicity and DOX-induced microbial dysbiosis by reducing the abundance of *Pseudomonadota* such as *Escherichia–Shigella*, *Dubosiella* and *Allobaculum*, along with increasing beneficial bacteria, especially *Muribaculaceae_unclassified*, *Ralstonia* and *Rikenellaceae* in the gut. Furthermore, to establish the direct relationship between alterations in gut microbiota and the protective effect of polyphenols on cardiotoxicity mediated by DOX, the test animals received a broad-spectrum antibiotics treatment to deplete intestinal microbiota. Interestingly, there was a pattern shift of *Bacillota* (formerly *Firmicutes*) and *Bacteroidota* (formerly *Bacteroidetes*) to *Pseudomonadota* (formerly *Proteobacteria*) in the gut microflora, and a great worsening of DOX-induced cardiotoxicity, evidenced by increased cardiac fibrosis and apoptosis, as well as reduced cardiac and mitochondrial function.

Another polyphenol of great interest is the isoflavone group. These are mainly found in soybeans, but also chickpeas, legumes, fava beans, pistachios, peanuts and other fruits and nuts. Many health benefits have been associated with the consumption of foods containing isoflavones due to their antioxidative diphenolic compounds. A high body of evidence has already reported a positive effect of isoflavones in cardiovascular diseases, especially concerning lipid abnormalities [139]. We highlight a single study which evaluated the pharmacologic use of Glabridin (GLA), an isoflavone derived from licorice root, in reducing DOX-induced cardiotoxicity through a link with the gut microbiota. In this experimental study [132], the consumption of GLA before DOX treatment attenuated DOX-induced cardiotoxicity through direct effects in the heart, such as the downregulation of pro-apoptotic proteins (Bax, cleaved-caspase 9 and cleaved-caspase 3), the upregulation of anti-apoptotic proteins (HAX-1 and Bcl-2) in the cardiac tissues, and modulation of colonic macrophages. GLA reduced the production of M1-like cytokines (IL-1β and TNF-α) and increased the production of M2-like cytokines (TGF-β and IL-10). Concomitantly, GLA modulated the DOX-induced dysbiosis of gut microbiota by decreasing the *Desulfovibrio* genus, a Gram-negative LPS-producing bacteria, improving the abundance of the Lactobacillus genus, which in turn exerts beneficial effects in the intestinal environment.

More recently, Zhao et al. investigated the effects of *Apocynum venetum*, a leaf extract containing bioactive compounds such as flavonoids and polysaccharides, in mice chronically treated with doxorubicin [135]. In this study, treatment with A. venetum improved ejection fraction and fractional shortening on echocardiogram, along with improvement in cellular structure and a reduction in myocardial fibrosis. Additionally, there were significant changes in gut bacterial genera with the treatment compared to doxorubicin-treated animals.

Although additional experimental approaches are still needed to better understand the underlying mechanisms associated with cardioprotective effects of polyphenols, an interesting potential preventive strategy is emerging to alleviate cardiotoxicity induced by DOX treatment.

### 7.2. Zinc (II)–Curcumin Complexes

Zinc is an essential trace element for human metabolism that is required by more than 300 enzymes, facilitating protein folding and helping to regulate gene expression [140]. Preclinical and clinical studies state that zinc may have a protective role in cardiovascular disease [141] and also suggest that zinc is essential for the intestinal commensal microflora inherent to the gut microbiome. Zinc supplementation could improve gut wall integrity, thus contributing to reduced translocation of bacteria and gut microbiome metabolites into the systemic circulation [142].

Additionally, zinc alone may be less effective than in combination with antioxidants, such as curcumin [108]. Curcumin is a polyphenol strongly known for its anticancer, antibiotic, anti-inflammatory and anti-aging properties [143]. One study evaluated the association of zinc with curcumin (ZnCM) to prevent DOX-induced cardiomyopathy through gut microbiota modulation. Wu et al. [108] showed that pretreatment with ZnCM supplementation improved gut microbiota composition by increasing the abundance of *Bacillota* and decreasing the abundance of *Bacteroidota* in DOX-treated rats, besides attenuating LPS levels in plasma and feces. Also, the degradation of the intestinal barrier integrity induced by DOX treatment was significantly prevented in ZnCM supplementation groups. All these structural and composition shifts in the gut microbiome were related to improvement in heart function, with a reduction in cardiomyocyte apoptosis and myocardial injury in doxorubicin-treated rats. Subsequently, horizontal feces were transferred from ZnCM-treated rats to normal matched-recipient rats and the cardioprotective and microbiota-modulating effects were preserved, corroborating the premise of an early causal link between gut microbial ecology and cardiotoxicity induced by DOX.

### 7.3. Emodin

Emodin is a natural anthraquinone that showed cardioprotective effects in a variety of cardiovascular diseases, including myocardial infarction, heart failure, diabetic cardiomyopathy and septical myocardial injury, and it appears to regulate the gut microbiota and maintain the intestinal structure in previous studies. Hu et al. reported that emodin reduced myocardial fibrosis, cardiomyocyte hypertrophy and myocardial disorganization while restoring the gut microbiota to levels close to normal, disrupted by doxorubicin treatment in mice [134]. Also, Dai et al. showed that emodin therapy enhanced cardiac function and reduced cardiomyocyte pyroptosis induced by doxorubicin in mice [144]. Similarly, He et al. reported a protective effect against doxorubicin-induced cardiotoxicity in H9c2 rat cardiomyocytes by inhibiting ferroptosis and oxidative stress [145].

### 7.4. Fecal Microbiota Transplantation

Fecal microbiota transplantation (FMT) has been studied as a medical therapeutic to correct gut dysbiosis by transferring stool from a healthy donor into another gastrointestinal tract [146]. Currently, FMT is only approved as a treatment of recurrent or refractory Clostridioides difficile infection; however, FMT has been used experimentally as a potential therapy targeting to treat of other conditions, including gastrointestinal, metabolic, neurological and cardiovascular diseases [33,146,147].

Recent evidence also suggested that FMT positively contributes to cardiac function by improving gut microbiome in DOX-treated mice. In one study [13], FMT treatment was able to reverse DOX-induced gut dysbiosis and ameliorated pathological gut changes such as goblet cell loss, the number of intestinal ulcers and lymphocyte cluster infiltration and loss of tight junction protein. It also decreased plasma endotoxin level when compared with DOX-alone-treated group. Concurrently, an improvement in heart function was observed in DOX-treated mice receiving FMT.

Moreover, Zhou et al. showed that FMT improved cardiac function and reduced cardiomyocyte vacuolization, fibrosis and apoptosis, and preserved the colonic mucosa thickness, the number of goblet cells and upregulated ZO-1 in mice treated chronically with doxorubicin [133]. Additionally, FMT reduced dynamin-related protein (DRP) and increased mitofusin 2 (MFN2), proteins related to mitochondrial fission and fusion, respectively, and improved the function of mitochondrial complexes I and III.

Undoubtedly, these observations show a clear heart–gut interaction, and the modulation of intestinal microbiota by FMT has a potential role to mitigate cardiomyopathy related to DOX treatment and may be a novel therapeutic strategy to alleviate chemotherapy.

### 7.5. Probiotics

Some studies have investigated the effect of probiotics, that are defined as live microbial food supplements [148], in improving gastrointestinal toxicity induced by chemotherapy. Xia et al. reported a reduction in mucositis severity in patients who received a probiotic cocktail and a less severe oral mucositis in rats treated with probiotics by downregulation of toll-like receptor-4 (TLR4) and restoration of zonula occludens-1 (ZO-1) and claudin-1 [149]. While numerous studies have focused on the use of probiotics to enhance the anti-tumor efficacy of doxorubicin [150,151,152], we found only two studies that have specifically evaluated the effects of probiotics on doxorubicin-induced cardiotoxicity. Stewart et al. did not find consistent results on cardiac function in rats treated with doxorubicin that received milk and kefir [153]. Abu-Elsaad et al. reported that rats treated with doxorubicin and provided with yogurt-enriched food exhibited lower levels of inflammatory cytokines and oxidative stress markers, along with enhanced cardiac contractility, but did not evaluate the composition of the microbiota [154].

### 7.6. Bacterial Membrane Protein Nanodrug

Recognizing the potential benefits of bacteria in maintaining intestinal barrier integrity, Li et al. synthesized an orally available membrane protein nanodrug derived from the bacterium *Akkermansia muciniphila*. *Amuc_1100*, an outer membrane protein, has been shown to replicate some of the beneficial functions of this bacterium [136]. It has been reported that *Amuc_1100* can reduce obesity, improve metabolic syndrome, alleviate ulcerative colitis, regulate immune responses and maintain the integrity of the gut barrier through its interaction with TLR2. In this study, the nanodrug improved doxorubicin-induced histological damage in the ileum, evidenced by reductions in villi shortening, increased crypt depth, and decreased inflammatory cell infiltration, along with reductions in LPS and zonulin levels in the serum. Concerning the microbiota, there were significant changes observed in both α and β-diversity. In regards to cardiac function, the treatment improved ejection fraction and fractional shortening, reduced levels of NT-proBNP, T troponin, and CKMB, decreased the area of cardiac fibrosis, and suppressed myocardial apoptosis.

## 8. Conclusions, Final Considerations and Future Perspectives

Numerous studies have explored the effects of various substances on the gut microbiota and their association with cardiovascular diseases. In contrast, only a limited number of small studies have investigated the potential relationship between doxorubicin, dysbiosis and cardiotoxicity. However, the scientific evidence linking the gut microbiota dysbiosis and the intestinal barrier disruption with the pathophysiology of doxorubicin-induced cardiotoxicity is increasing in the recent years.

Polyphenols, zinc (II)-curcumin complexes, emodin, fecal microbiota transplantation, probiotics and bacterial membrane protein nanodrug alter the gut microbiota, improving cardiac function by reducing inflammation, oxidative stress, mitochondrial dysfunction and apoptosis. Further research targeting microbiota and intestinal permeability is required aiming to elucidate the precise pathophysiology of the gut–heart axis and to identify effective drugs or substances that may treat or mitigate doxorubicin-induced cardiotoxicity.

## Figures and Tables

**Table 1:** Therapeutic strategies regarding modulation of microbiota on DOX-induced cardiotoxicity.

Study	Investigated Compound	Model	General Effects	Cardiac Effects	Microbiota and Intestinal Effects	Ref.
Wu et al., 2020	*Arctium lappa* L.	Mice	↓ OS, ↓ NO, ↓ TNF-α, ↓ casein kinase, ↓ LDH	Amelioration of morphological damage	↓ Species richness and diversity of microbial community ↑ Lactobacillaceae, ↑ Ruminococcaceae, ↑ Roseburia ↓ Proteobacteria, ↓ Enterococcus, ↓ Erysipelatoclostridium ↓ Escherichia-Shigella	[131]
Lin et al., 2021	Yellow wine	Rats	↓ LDH, ↓ CK-MB, ↓ mitochondrial damage, ↓ TNF- α, IL-1β, IL-6, IL-8	Improvement of LVEF, LVSF, LVPW ↓ collagen deposition ↓ cardiomyocite size, ↓ myocardial apoptosis	↓ Bacterial species richness, ↓* Escherichia–Shigella*, ↓* Dubosiella*, ↓* Allobaculum* ↑* Muribaculaceae*, ↑* Ralstonia*, ↑* Rikenellaceae*	[107]
Huang et al., 2019	Glabridin	Rats	↓ IL-1β, ↓TNF-α, ↑ TGF-β, ↑ IL-10	↓ Bax, ↓ cleaved-caspase 9, ↓ cleaved-caspase 3 ↑ HAX-1, ↑ Bcl-2	↓* Desulfovibrio* genus, ↑* Lactobacillus* genus	[132]
Wu et al., 2019	Zinc (II)-curcumin	Rats	↓ CK, ↓ CK-MB, ↓ LDH, ↓ INF-γ, ↓ IL-6, ↓ TNF-α, ↓ IL-1β, ↓ MCP-1	↓ Cardiac apoptosis, ↑ LVEDP, ↓ fibrosis	↑* Firmicutes*, ↓* Bacteroidetes*, ↓ LPS, ↑ ZO-1, ↑ occludins, ↓ inflammatory cells, ↑ crypt depth, ↑ TJ, ↑ goblet cells	[108]
An et al., 2020	Fecal transplantation	Mice	↓ NOX-2, ↓ TLR-2, ↓ IL-1β	↑ LVFE, ↑ FS, ↓ perivascular and intersticial fibrosis	↑ Goblet cells, ↓ intestinal ulcers, ↓ lymphocyte infiltration, ↑ TJ, ↑ ZO-1, ↓ LPS, ↑ microbiota richness	[13]
Zhou et al., 2024	Fecal transplantation	Mice	↓ MDA, ↑ SOD, ↓ Drp, ↑ MFN-2, improved mitochondrial complexes I and III, ↑ Nrf2	↑ LVFE, ↑ FS, ↓ collagen deposition, ↓ vacuolization, ↓ apoptosis	Alteration of p_Proteobacteria, c_Gammapro-teobacteria, o_Bacteroidales, and p_Bacteroidota	[133]
Hu et al., 2023	Emodin	Mice	↑ Nrf2, ↑ HO-1, ↑ NQO1	↓ Myocardial fibrosis, ↓ hypertrophy, and ↓ disorganization	↑* Bacteroidota*, ↓* Verrucomicrobiota*	[134]
Zhao et al., 2023	*Apocynum venetum* leaf extract	Mice	↓ cardiac apoptosis, ↓ BNP	↑ Ejection fraction, ↑ fractional shortening, ↓ myocardial fibrosis	↑ *Escherichia−Shigella*, ↑ *Akkermansia*, ↑ *Bacteroides*, ↑ *Clostridium*, ↑ *Ruminococcus*, ↑ *Enterobacter*, ↑ *Anaerotruncus*, ↑ *Enterorhabdus*, ↑ *Faecalibaculum*, ↑ *Romboutsia*, and ↑ *Halomonas*	[135]
Li et al., 2024	Bacterial membrane protein nanodrug	Mice	↓ IL-1β, IFN-γ, TNF-α, NT-proBNP, T troponin, CKMB	↑ Ejection fraction, ↑ fractional shortening, ↓ myocardial fibrosis, ↓ myocardial apoptosis	↑ crypt depth, ↓ inflammatory cell infiltration, ↓ reduced LPS, ↓ zonulin ↑ short-chain fatty acid-producing bacteria, ↑ butyrate and pentanoic acids, changes in α and β-diversity	[136]

↓: decrease, ↑: increase, OS: oxidative stress, NO: nitric oxide, TNF-α: tumor necrosis factor alpha, LDH: lactate dehydrogenase, CK-MB: creatine kinase-myocardial band, CK: creatine kinase, IL-1β: interleukin 1β, IL-6: interleukin 6, IL-8: interleukin 8, LVEF: left ventricular ejection fraction, LVSF: left ventricular shortening fraction, LVPW: left ventricule posterior wall thickness, TGF-β: transforming growth factor β, IL-10: interleukin 10, Bax: bcl-2-like protein 4, HAX-1: HCLS1-associated protein X, LPS: lipopolysaccharide, ZO-1: zonula occludens 1, MDA: malonaldehyde, SOD: superoxide dismutase, Drp: dynamin-related protein, MFN-2: mitofusin 2, TJ: tight junction, LVEDP: left ventricular end diastolic pressure, INF-γ: interferon γ, MCP-1: monocyte chemoattractant protein-1, FS: fractional shortening, NOX-2: NADPH oxidase 2, TLR-2: toll-like receptor 2, Nrf2: Nuclear factor erythroid 2-related factor 2, HO-1: heme oxygenase, NQO: NAD(P)H:quinone oxidoreductase 1, BNP: brain natriuretic peptide.

## Data Availability

No new data were created or analyzed in this study.
